# Assessing the prognostic value of tumor-infiltrating CD57+ cells in advanced stage head and neck cancer using QuPath digital image analysis

**DOI:** 10.1007/s00428-022-03323-6

**Published:** 2022-04-22

**Authors:** Emma J. de Ruiter, Sangeeta K. Bisheshar, Reinout H. de Roest, Frederik W. R. Wesseling, Frank J. P. Hoebers, Mari F. C. M. van den Hout, C. René Leemans, Ruud H. Brakenhoff, Remco de Bree, Chris H. J. Terhaard, Stefan M. Willems

**Affiliations:** 1 Department of Pathology, University Medical Center Utrecht, Heidelberglaan 100, 3584 CX Utrecht, The Netherlands; 2 Department of Pathology, University Medical Center Utrecht, H04.312, 3508 GA Utrecht, The Netherlands; 3 Department of Pathology, University Medical Center Groningen, Groningen, The Netherlands; 4 Department of Otolaryngology/Head and Neck Surgery, Amsterdam University Medical Center, Vrije Universiteit Amsterdam, Cancer Center Amsterdam, Amsterdam, The Netherlands; 5 Department of Radiation Oncology (MAASTRO), GROW – School for Oncology and Developmental Biology, Maastricht University Medical Centre, Maastricht, The Netherlands; 6 Department of Pathology, Maastricht University Medical Center, Maastricht, The Netherlands; 7 Department of Head and Neck Surgical Oncology, University Medical Center Utrecht, Utrecht, The Netherlands; 8 Department of Radiotherapy, University Medical Center Utrecht, Utrecht, The Netherlands

**Keywords:** Head and neck squamous cell carcinoma (HNSCC), Tumor-infiltrating lymphocytes (TILs), NK cells, Prognostic biomarkers, Digital pathology, QuPath

## Abstract

This study aimed to assess the prognostic value of intratumoral CD57+ cells in head and neck squamous cell carcinoma (HNSCC) and to examine the reproducibility of these analyses using QuPath. Pretreatment biopsies of 159 patients with HPV-negative, stage III/IV HNSCC treated with chemoradiotherapy were immunohistochemically stained for CD57. The number of CD57+ cells per mm^2^ tumor epithelium was quantified by two independent observers and by QuPath, software for digital pathology image analysis. Concordance between the observers and QuPath was assessed by intraclass correlation coefficients (ICC). The correlation between CD57 and clinicopathological characteristics was assessed; associations with clinical outcome were estimated using Cox proportional hazard analysis and visualized using Kaplan-Meier curves. The patient cohort had a 3-year OS of 65.8% with a median follow-up of 54 months. The number of CD57+ cells/mm^2^ tumor tissue did not correlate to OS, DFS, or LRC. N stage predicted prognosis (OS: HR 0.43, *p* = 0.008; DFS: HR 0.41, *p* = 0.003; LRC: HR 0.24, *p* = 0.007), as did WHO performance state (OS: HR 0.48, *p* = 0.028; LRC: 0.33, *p* = 0.039). Quantification by QuPath showed moderate to good concordance with two human observers (ICCs 0.836, CI 0.805–0.863, and 0.741, CI 0.692–0.783, respectively). In conclusion, the presence of CD57+ TILs did not correlate to prognosis in advanced stage, HPV-negative HNSCC patients treated with chemoradiotherapy. Substantial concordance between human observers and QuPath was found, confirming a promising future role for digital, algorithm driven image analysis.

**Supplementary Information:**

The online version contains supplementary material available at 10.1007/s00428-022-03323-6.

## Introduction

Head and neck squamous cell carcinomas (HNSCC) comprise a heterogeneous group of malignancies originating from the mucosa of the nasal and oral cavity, nasopharynx, oropharynx, hypopharynx, and larynx [1]. Approximately 60% of HNSCC patients have an advanced stage of disease at the time of diagnosis [2]. Treatment of advanced stage HNSCC requires a multidisciplinary approach, using combinations of surgery, radiotherapy, chemotherapy, and targeted therapies. Despite numerous developments in therapeutic options, the prognosis of head and neck cancer patients remains poor: 5-year survival rates range from 56 to 62% for all clinical stages combined and 15–50% of HNSCC patients face locoregional failure, strongly contributing to morbidity and mortality [3–6]. Availability of reliable biomarkers predicting which patients are at risk for recurrent disease is essential for optimizing treatment for individual patients.

In recent decades, it has become clear that tumor behavior and response to therapy are largely influenced by the interaction between tumor cells and their tumor microenvironment (TME), and numerous studies have examined the possibility to employ the immune system in cancer treatment and diagnostics. Natural killer (NK) cells have recently become an increasingly investigated target. Because of their major histocompatibility complex (MHC)-unrestricted cytotoxic ability, cytokine production, and immunologic memory, NK cells are considered to be a distinct group of anti-tumor response cells, combining functions of both the innate and adaptive immune system [7]. Agents specifically targeting inhibitory receptors on the surface of NK cells, such as monalizumab and lirilumab, have shown mild success rates in cancer treatment [8]. Also checkpoint inhibitors that are already FDA-approved, such as certain PD-1/PD-L1 and CTLA-4 inhibitors, enhance NK cell-mediated cytotoxicity [9–11]. Furthermore, NK cells are able to enhance the effect of therapy with monoclonal antibodies through antibody-dependent cell-mediated cytotoxicity (ADCC) through cross-linking with CD16 [12, 13].

CD57 is an immune marker expressed on the cell membrane of differentiated T cells and NK cells [14]. In T cells, CD57 is expressed on cells with a terminally differentiated phenotype, characterized by increased cytotoxicity and impaired proliferative capability [15]. CD57+ NK cells comprise a subset of mature NK cells with increased cytotoxicity, characterized by upregulation of CD16 and degranulation markers CD107a, granzyme B, and perforin [16]. As an indicator for maturation and increased cytotoxicity of immune effector cells, CD57 might be an interesting biomarker in the anti-tumor immune response. In this study, we investigate its prognostic value in a cohort of HPV-negative, advanced stage HNSCC patients treated with chemoradiotherapy. In the quantitative evaluation of tissue-based biomarkers, digital image analysis plays an increasingly important role. The use of digital pathology has numerous advantages for research, education, and diagnostics. Currently, scanning technology, image processing, and image understanding algorithms are coming together to fully put digital image analysis into practice [17–20]. Tissue-based biomarker studies could strongly benefit from digital image analysis. They usually involve manual scoring of immunohistochemically stained tissue slides or tissue microarrays (TMAs), a laborious approach conducted by pathologists or researchers, with inevitable inaccuracies due to intraobserver and interobserver variability [21]. The availability of robust digital image analysis software could reduce time and costs and at the same time increase accuracy and reproducibility. QuPath is an open source software platform for digital pathology and image analysis. Several studies assessed the robustness and reproducibility of QuPath, generally confirming its usability for tissue-based biomarker studies [22].

The aim of this study was twofold: first to assess the prognostic value of CD57+ tumor infiltrating lymphocytes (TILs) in head and neck tumors; and second to investigate the reproducibility of these analyses using QuPath digital image analysis.

## Methods

### Patients and clinical data

This study was conducted using a consecutive, retrospective cohort of HNSCC patients, which was partly described before [23]. The cohort consisted of patients that were treated at the University Medical Center (UMC) Utrecht, the Amsterdam University Medical Center (location VUmc), and the Maastricht University Medical Center between January 2009 and December 2014. The following inclusion criteria were applied: (1) stage III or IV, HPV-negative oropharyngeal, hypopharyngeal, and laryngeal squamous cell carcinoma; (2) treatment with radiotherapy with concomitant cisplatin or carboplatin with curative intent; and (3) availability of tumor tissue and clinical data on survival outcome. Patients treated with surgical resection of the tumor, or having distant metastases at diagnosis, previous treatment with radiotherapy to the head and neck area, or a prognosis-affecting second primary tumor or prior malignancy were excluded.

For each patient, the following clinicopathological data were collected: age, sex, performance status, comorbidity, prior malignancies, tobacco and alcohol usage, tumor localization, tumor stage (TNM-7), T stage, N stage, total radiation dose, and total chemotherapy dose. Comorbidity was scored using the Adult Comorbidity Evaluation-27 (ACE-27) [24]. Performance status was scored using the WHO classification [25].

### Treatment protocol

Standard treatment regimen existed of a total radiation dose of 70 Gy to the primary tumor and involved lymph nodes in 35 fraction of 2 Gy, and a total dose of 46–57.75 Gy on the elective lymph nodes, in combination with cisplatin in a total dose of 300 mg/m^2^ body surface area in three divided doses every 3 weeks.

### Tissue microarray construction and immunohistochemistry

Formalin-fixed, paraffin-embedded (FFPE) pretreatment biopsies of all included patients were collected in a tissue microarray (TMA) as described before [23]. In short, sections of the FFPE blocks were stained with hematoxylin and eosin (H&E) and assessed by a dedicated head and neck pathologist (SMW) to mark representative tumor regions. For each patient, three 0.6-mm tissue cores were obtained randomly from the assigned area of the FFPE blocks and collected in a TMA. The TMA blocks were cut into 4-μm sections, which were immunohistochemically stained for CD57 (NK1; 1:20; Novocastra). Stainings were performed using a Ventana Bench Mark XT Autostainer (Ventana Medical Systems, Tucson, AZ, USA).

### HPV detection

Only HPV-negative tumors were included in this study. Oropharyngeal tumors were considered HPV-negative if less than 70% of tumor cells stained positive for p16^INK4a^ by immunohistochemistry (JC8, 1:1200, Immunologic). P16-positive tumors were tested for the presence of HPV-DNA by PCR and were excluded when high-risk HPV-DNA was detected [23, 26]. All hypopharyngeal and laryngeal tumors were considered HPV-negative.

### Immunohistochemical analysis by two observers

**Fig. 1:**
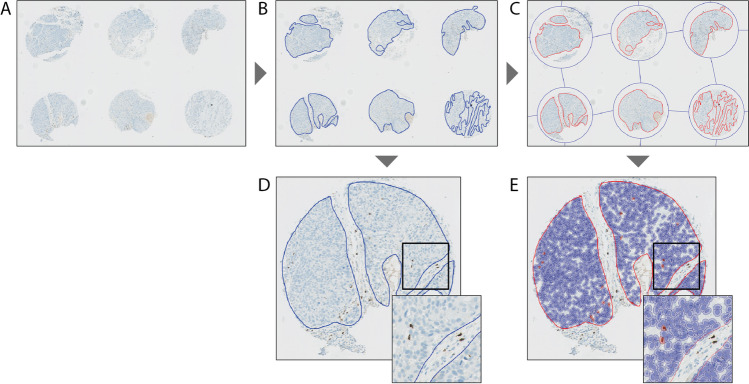
Immunohistochemical analysis of the presence of CD57+ cells. (**A**) Stained sections of the TMA were digitized. (**B**) For each TMA core, the tumor epithelium was digitally annotated. (**C**) TMA cores were identified using QuPath’s TMA dearranger function and tumor annotations were imported in QuPath. (**D**) Within the annotated area, the number of CD57-positive cells were independently scored by two head and neck researchers. (**E**) Positive cells within the annotated area were counted using the *Positive Cell Detection* command.

Stained sections of the TMA were digitized using Aperio Scanscope XT slide scanner at a magnification of 40× (Fig. 1A). For each TMA core, the tumor epithelium was digitally annotated by a dedicated head and neck pathologist (SMW) using Aperio ImageScope 12.1 (Fig. 1B). Within this annotated area, the number of CD57+ TILs was independently scored by two head and neck cancer researchers (EDR and SB), who were blinded for clinical outcome (Fig. 1D). The number of intratumoral CD57+ TILs per mm^2^ tumor was calculated by dividing the summed number of the three corresponding TMA cores by the total tumor area of the three cores. For the observer score, the average of the two scores per core was used. Tumors were considered eligible for inclusion if at least two TMA cores were assessable and if the total annotated tumor area was more than 0.1 mm^2^.

### Immunohistochemical analysis by QuPath

TMA cores were identified using QuPath’s TMA dearranger function (QuPath version 0.1.6). The annotations used for the manual scoring were imported into QuPath (Fig. 1C). Before analysis, color deconvolution was applied using the *Estimate Stain Vectors* command on a representative TMA core; the same vectors were used across all TMAs. Positive cells within the annotated area were counted using the *Positive Cell Detection* command (Fig. 1E). The total number of positive cells within the annotated area was obtained. A script was generated and run on all individual TMA slides to automate the detection process

### Outcome measures

The association between the number of CD57+ TILs/mm^2^ and overall survival (OS), disease-free survival (DFS), and locoregional control (LRC) was investigated by a Cox proportional hazards model. OS was defined as the number of days between the first day of treatment and the date of death, DFS as the number of days between the first day of treatment and the date of recurrence of disease or the date of death, and LRC as the number of days between the first day of treatment and the date of local or regional recurrence. Patients without an event were censored at the date of their last visit to the clinic.

### Statistical analysis

Correlations between CD57 and clinical variables were assessed by Mann-Whitney *U* tests for dichotomous clinical variables, Kruskal-Wallis tests for clinical variables stratified in more than two groups, and Spearman correlation for continuous clinical variables. Correlations with OS, DFS, and LRC were assessed using Cox proportional hazards regression in Rstudio (version 1.1.456) using the survival and survminer packages. In order to perform the regression analysis, CD57+ cell counts were log transformed by taking their log_2_. The predictive value of CD57 was visualized by a Kaplan-Meier curve comparing tumors with high and low CD57+ cell count stratified by the median value; hazard ratio (HR) and *p*-value accompanying the Kaplan-Meier curves were calculated using log rank tests. Intraclass correlation coefficients (ICC) between different TMA cores from the same patient were calculated using SPSS (SPSS statistics 26.0.0.1) based on a mean-rating (*k* = 3), absolute-agreement, 2-way mixed effects model [27]. Concordance between the observers’ results and the results generated using QuPath was estimated by intraclass correlation coefficients (ICC). ICCs between the scores of individual TMA cores were calculated based on a mean-rating (*k* = 2), absolute-agreement, 2-way random effects model using Rstudio [27]. In order to assess the correlations with clinical variables and survival, and ICC between different TMA cores from the same patient, the average score of the two observers was used.

## Results

### Patient characteristics

The patient cohort consisted of 159 patients, among which 72 oropharyngeal, 56 hypopharyngeal, and 31 laryngeal cancer patients, with a 3-year OS of 65.8%, and a median duration of follow-up of 54 months. Clinical characteristics of the patient cohort are summarized in Table 1.

**Table 1:** Patient characteristics

Age (years)	Mean (SD)	59.2	(6.2)
Sex	Male Female	98 61	(61.6%) (38.4%)
WHO performance status	0 1 2 Unknown	39 98 5 19	(23.9%) (61.6%) (3.1%) (11.3%)
ACE-27 comorbidity score	None (0) Mild (1) Moderate (2) Severe (3)	54 78 26 1	(34.0%) (49.1%) (16.4%) (0.6%)
Prior malignancy		12	(7.5%)
	HNSCC Other	3 9	(1.9%) (5.7%)
Tobacco use	Current Former Never Unknown	114 38 6 1	(71.7%) (23.9%) (3.8%) (0.6%)
Smokackyears	Mean (SD)	40.0	(17.9)
Alcohol	Current 1–3/day ≥4/day Former Never Unknown	107 53 54 34 16 2	(67.3%) (33.3%) (34.0%) (21.4%) (10.1%) (1.3%)
Tumor location	Oropharynx Hypopharynx Larynx	72 56 31	(45.3%) (35.2%) (19.5%)
T stage	T1 T2 T3 T4a T4b	4 29 59 53 14	(2.5%) (18.2%) (37.1%) (33.3%) (8.8%)
N stage	N0 N1 N2a N2b N2c N3 Unknown	22 22 11 52 45 6 1	(13.8%) (13.8%) (6.9%) (32.7%) (28.3%) (3.8%) (0.6%)
TNM stage	III IVa IVb	30 111 18	(18.9%) (69.8%) (11.3%)
Chemotherapy completed	Yes Switch No	117 16 26	(73.6%) (10.1%) (16.4%)
Treatment outcome	No recurrence Residue/recurrence Locoregional Distant	104 55 41 30	(65.4%) (34.6%) (25.8%) (18.9%)

All patients were treated with radiotherapy in combination with a platinum-based chemotherapeutic agent. Most patients received cisplatin; five patients were treated with carboplatin instead of cisplatin. A total of 16 patients were initially treated with cisplatin but switched to carboplatin due to adverse events. In total, 26 patients discontinued treatment after two doses of cisplatin, thereby receiving a total dose of 200 mg/m^2^ body surface area.

### Immunostaining of CD57 on pretreatment biopsies and correlation with clinicopathological characteristics

The median number of CD57+ TILs in this cohort was 17.1 cells/mm^2^ (interquartile range: 8.8–40.3). The median of the log_2_ transformed number was 2.9 (interquartile range: 2.3–3.7). Concordance between TMA cores from the same patients was moderate to good (ICC: 0.78, 95% confidence interval (CI): 0.69–0.84). No correlations between CD57 and clinicopathological characteristics were observed (Table 2).

**Table 2:** Correlation between CD57 and clinicopathological characteristics. For each subgroup, the median CD57 count, interquartile ranges, and *p*-values are displayed

	CD57	*p*
Age		
	*r* = 0.135	0.09
Sex		
Male Female	20.8 [9.0–38.7] 15.9 [4.1–41.3]	0.20
ACE-27		
None/mild Moderate/severe	17.0 [8.8–40.6] 20.0 [8.8–34.4]	0.96
WHO		
<2 ≥2	21.0 [5.4–77.2] 16.1 [8.8–36.6]	0.25
Tumor location		
Oropharynx Hypopharynx Larynx	16.1 [8.2–40.6] 20.1 [5.8–40.9] 11.9 [9.0–25.7]	0.66
T stage		
T1–3 T4	18.0 [9.1–40.8] 16.8 [3.1–40.3]	0.27
N stage		
N0–1 N2–3	19.8 [8.9–39.9] 16.9 [8.6–41.6]	0.99

### Correlation between CD57+ cells and treatment outcome

The outcome of all survival analyses is shown in Table 3. No significant correlations were found between the presence of CD57+ TILs and OS, DFS, or LRC. Kaplan-Meier curves visualizing the prognostic value of CD57 are shown in Supplementary Fig. 1. Due to lack of correlation between CD57+ cell count and survival data, no multivariate analysis was performed.

**Table 3:** Correlation between intratumoral CD57+ and treatment outcome. Hazard ratios (HR), 95% confidence interval (95%CI), and *p*-values are displayed

Marker	Comparison	No. of cases	HR	95%CI	*p*-value
Overall survival					
CD57	Per 1 increase (log_2_)	159	0.96	(0.81–1.15)	0.68
Tumor location	Larynx	159	Ref		
	Oropharynx		1.96	(0.97–3.95)	0.073
	Hypopharynx		1.22	(0.58–2.57)	0.61
T stage	T1–3 vs T4	159	0.76	(0.47–1.22)	0.26
N stage	N0–1 vs N2-3	159	**0.43**	**(0.23–0.80)**	**0.008**
Age	Per year increase	159	0.99	(0.96–1.03)	0.73
Sex	Male vs female	159	1.35	(0.81–2.25)	0.28
ACE-27	<2 vs ≥2	159	0.84	(0.45–1.58)	0.60
WHO	<2 vs ≥2	141	**0.48**	**(0.25–0.93)**	**0.028**
Disease-free survival					
CD57	Per 1 increase (log_2_)	159	0.99	(0.84–1.17)	0.91
Tumor location	Larynx	159	Ref		
	Oropharynx		1.71	(0.90–3.26)	0.11
	Hypopharynx		1.27	(0.64–2.50)	0.49
T stage	T1–3 vs T4	159	0.86	(0.55–1.35)	0.52
N stage	N0–1 vs N2–3	159	**0.41**	**(0.23–0.74)**	**0.003**
Age	Per year increase	159	1.00	(0.96–1.03)	0.85
Sex	Male vs female	159	1.30	(0.82–2.07)	0.27
ACE-27	<2 vs ≥2	159	0.99	(0.54–1.79)	0.97
WHO	<2 vs ≥2	141	0.60	(0.33–1.07)	0.083
Locoregional control					
CD57	Per 1 increase (log_2_)	159	1.17	(0.92–1.49)	0.19
Tumor location	Larynx	159	Ref		
	Oropharynx		2.19	(0.83–5.78)	0.11
	Hypopharynx		1.36	(0.48–3.85)	0.57
T stage	T1–3 vs T4	159	0.78	(0.43–1.50)	0.48
N stage	N0–1 vs N2–3	159	**0.24**	**(0.085–0.68)**	**0.007**
Age	Per year increase	159	1.00	(0.95–1.05)	0.88
Sex	Male vs female	159	0.96	(0.53–1.94)	0.96
ACE-27	<2 vs ≥2	159	1.76	(0.63–4.95)	0.29
WHO	<2 vs ≥2	141	**0.33**	**(0.12–0.94)**	**0.039**

### Correlation between clinicopathological characteristics and treatment outcome

The clinical variables that correlated to treatment outcome in our cohort were N stage and WHO performance status. N0 and N1 patients showed significantly better OS (HR 0.43, 95%CI 0.23–0.80, *p* = 0.010), DFS (HR 0.41, 95%CI 0.23–0.74, *p* = 0.004), and LRC (HR 0.24, 95%CI 0.085–0.68, *p* = 0.007) than N2 and N3 patients. Patients with a WHO performance of 0 or 1 showed significantly better OS (HR 0.48, 95%CI 0.25–0.93, *p* = 0.028) and LRC (HR 0.33, 95%CI 0.12–0.94, *p* = 0.034) than patients with a WHO performance status of 2 or higher.

### Concordance between observers and QuPath

**Fig. 2:**
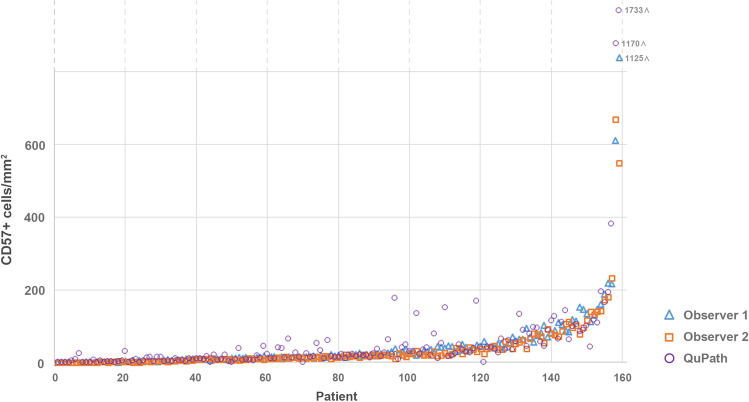
Visualization of the concordance between the two observers and QuPath. Plotted are the mean CD57+ cell counts per patient. Patients are ordered by the mean CD57+ cell counts of the two observers

Concordance between the two observers was excellent (ICC 0.924, CI 0.907–0.937). Concordance between the two observers and QuPath was moderate to good (ICC QuPath versus observer 1: 0.836, CI 0.805–0.863; ICC QuPath versus observer 2: 0.741, IC 0.692–0.783). Concordance between observers and QuPath on a patient level is visualized in Fig. 2, which shows the mean CD57+ cell count for each individual patient sorted by mean CD57+ cell count of the two observers. Discrepancies between QuPath and the two observers were mainly observed in TMA cores with profound background staining, artifacts, or multiple positive cells clustering together.

## Discussion

In this study, we assessed the prognostic value of CD57+ TILs in pretreatment biopsies of HNSCC patients using QuPath’s open source software platform for digital pathology and image analysis.

CD57 is an immune marker expressed on the cell membrane of differentiated T cells and NK cells. CD57 expression on NK cells characterizes a mature cell subset with increased cytotoxicity, linked to enhanced tumor surveillance. The presence of peripheral or intratumoral CD57+ NK cells has been associated to better outcomes in several cancer types [28]. Also in HNSCC, a meta-analysis of Bisheshar et al. showed that general NK cell markers CD56 and CD57 as well as some activating NK cell receptors correlated with better overall survival [29]. The clinical implications of CD57 expression on T cells seem to be more ambiguous. CD57+ T cells comprise a subset of senescent T cells with preservation of potent effector functions, however seeming incapable to inhibit the growth of malignant cells: accumulation of CD8+CD57+ TILs in the peripheral blood was associated to decreased survival in renal cell carcinoma, melanoma, and gastric carcinoma [14, 30–32]. The role of CD57+ T cells in the TME remains unclear. In our patient cohort, no correlation was found between the presence of CD57+ TILs in HNSCC and OS, DFS, or LRC. These results differ from the literature so far: several studies assessed the prognostic value of CD57 in HNSCC, and a meta-analysis showed that the accumulation of CD57+ TILs was correlated to a favorable prognosis in HNSCC patients [29, 33–38]. However, most studies used small patient cohorts with a high heterogeneity regarding tumor stage, location, or treatment modality. Besides, none of the studies accounted for HPV-status in their analyses.

Karpathiou et al. investigated the prognostic role of CD57 in a patient cohort most similar to ours in terms of tumor stage and location. They did find the presence of CD57+ TILs to be prognostically favorable, especially when located intraepithelially or at the tumor border [35]. However, almost all patients included in their study were surgically treated, often in combination with (neo)adjuvant chemotherapy or chemoradiotherapy, while all patients in our study were treated by definitive chemoradiotherapy only. It was shown in some cancer types that (chemo) radiotherapy affects the tumor microenvironment and is able to enhance the anti-tumor immune response, which could mean that the pre-treatment composition of the tumor microenvironment is of less importance in non-surgically treated patients [39–42]. However, several studies did show an association between the pre-treatment presence of immune cells and (chemo) radiotherapy outcome [43–45].

Another possible explanation for the difference between the results of this study and the literature is that we exclusively included HPV-negative tumors. None of the studies mentioned above accounted for HPV status in their analyses, although several of them included a substantial number of oropharyngeal tumors. This is remarkable, because HPV-positive and HPV-negative tumors are considered as different disease entities [46, 47]; and, more importantly, HPV-positive tumors are associated with both a better prognosis and a more immunogenic tumor microenvironment [48–50]. Therefore, HPV status could be a confounder if not corrected for and might explain the lack of prognostic value of CD57 in our cohort. Investigating the difference in CD57+ TILs between HPV-positive and HPV-negative tumors, and their relation with prognosis, would contribute to the understanding of the prognostic value of CD57 in both subtypes of HNSCC. Furthermore, we only investigated the prognostic value of the presence of intratumoral CD57+ and not of CD57+ TILs in the tumor stroma. This might have led to differences with the literature, as some studies report on CD57 in the stroma or tumor border only, or in all compartments combined. However, some studies specifically report a correlation between intratumoral CD57+ TILs in the tumor nests and prognosis.

Furthermore, in this study, a TMA was used instead of whole tissue slides. It has been shown that immune cells can be distributed unequally through the TME, suggesting that intratumor heterogeneity could play a role in the results of this study. However, three cores were taken per tumor specimen, which accounts for heterogeneity within the tumor biopsy [51]. A bigger restraint might be the limitation in the usage of patient material in the first place. As all patients in this cohort were treated with primary chemoradiotherapy, only small pre-treatment biopsies were available for research. It has to be noted that this does not only apply to research, but has to be taken into consideration in diagnostics as well.

Lastly, an important point of discussion is the use of CD57 as single marker in assessing TILs in the TME. This obviously has disadvantages, most importantly the inability to discriminate between specific immune cell subsets. However, single-marker immunohistochemistry has important advantages as well: it is relatively easy to establish, to perform, and to analyze, especially compared to double or multiplex immunohistochemistry or immunofluorescence. This makes single markers more reproducible and accessible, which we believe are important requirements for prognostic and predictive biomarkers to be used in clinical practice. Furthermore, many studies present research on single markers, which makes it easier to compare results to the current literature. Nonetheless, differentiating between NK cells and T cells might be important because of their possibly opposing functions and this could be an explanation for our negative findings.

The second aim of this study was to assess the reliability of the quantification of immunohistochemically stained immune cells using digital pathology and image analysis platform QuPath. In this study, it was shown that QuPath’s positive cell detection function could easily identify CD57+ TILs in previously annotated tumor tissue. Observer scores were highly concordant, which is supported by Fig. 2. Concordance between the human observers and QuPath was moderate to good. As displayed in Fig. 2, QuPath had some outliers compared to the human observers. The largest deviations were observed in TMA cores with profound background staining, artifacts, or multiple positive cells clustering together. Manual selection of representative regions of interest could optimize the software’s performance and accuracy.

In conclusion, this study did not provide evidence for a prognostic role of the presence of intratumoral CD57+ TILs in HNSCC. Furthermore, our results confirm a promising future role for digital, algorithm-driven image analysis of immunohistochemically stained tissue slides in both research and diagnostics.

## Supplementary Information

ESM 1 (PDF 172 kb)

## Declarations

For this study, only anonymous archival leftover pathology material was used. Therefore, no informed consent is required according to Dutch legislation (www.fedora.org), as the use of redundant tissue for research purposes is part of the standard treatment agreement with patients in our center [52]. The use of anonymous archival leftover material was approved by the Biobank Research Ethics Committee of the UMC Utrecht (protocol number 18-233).
