# Supplementary material for: Assessing the prognostic value of tumor-infiltrating CD57+ cells in advanced stage head and neck cancer using QuPath digital image analysis
Source: Virchows Arch. 2022 Apr 22;481(2):223–31. doi: 10.1007/s00428-022-03323-6 (PMC9343309; doi:10.1007/s00428-022-03323-6)
Supplement: Supplementary file 1 — (PDF 172 kb) [file 428_2022_3323_MOESM1_ESM.pdf]

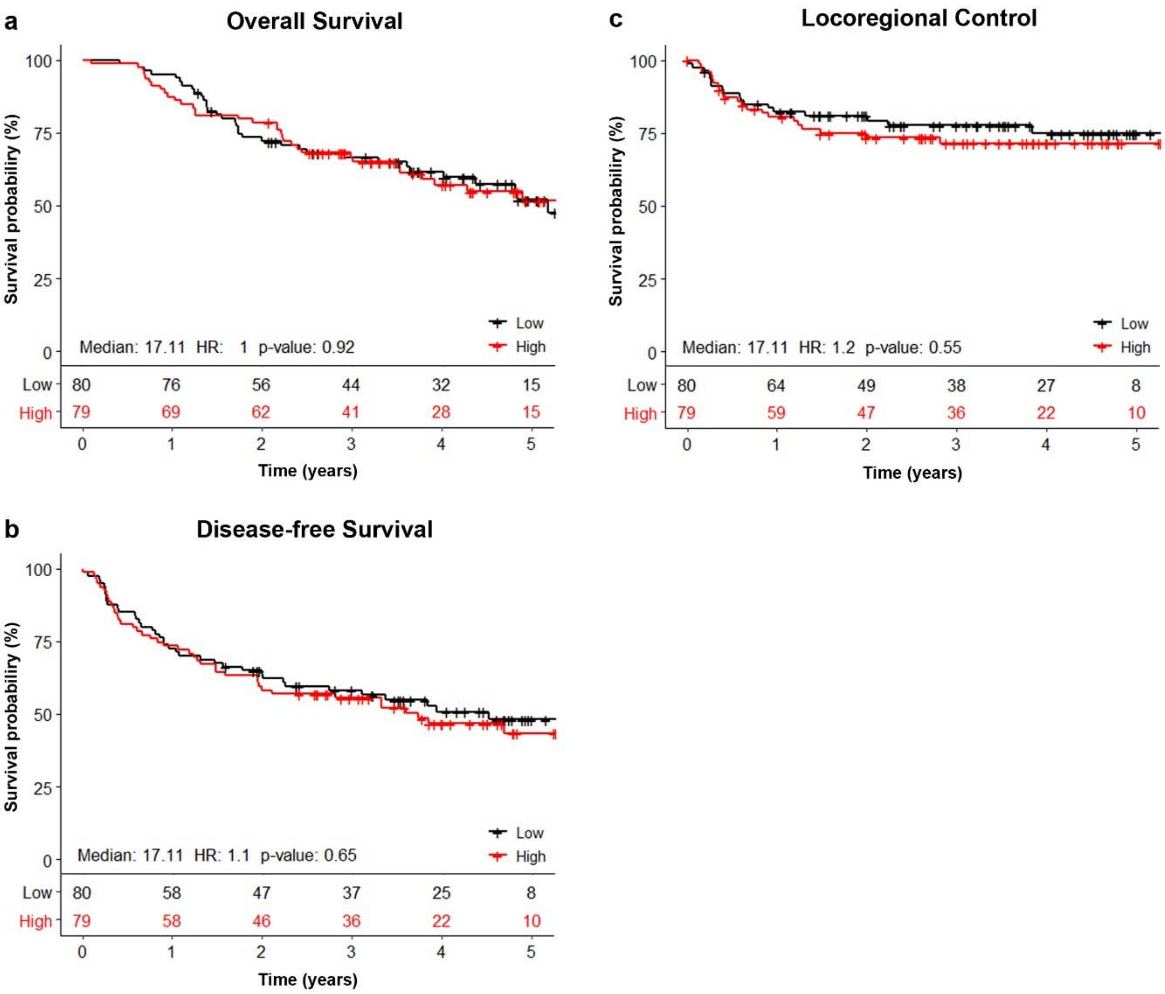

Supplementary figure 1: Kaplan-Meier curves visualizing the association between the number of CD57+ cells in the tumor epithelium and OS (**a**), DFS (**b**), and LRC (**c**). Data were dichotomized by the median CD57+ cell count. No association was found between CD57 and OS, DFS, or LRC.

Comparison	ICC (95% CI)
**Observer1 vs Observer2**	0.924 (0.907-0.937)
**Observer1 vs QuPath**	0.836 (0.805-0.863)
**Observer2 vs QuPath**	0.741 (0.692-0.783)

Supplemetary table 1: Concordance between the two observers and QuPath. Concordance was estimated by Intraclass Correlation Coefficients (ICC) between the scores of individual TMA cores based on a mean-rating (k = 2), absolute-agreement, 2-way random-effects model.
